# Massive Pericardial Effusion in a 14-Year-Old Girl with Mild Fatigue and Neck Pain

**DOI:** 10.3390/pediatric14010002

**Published:** 2022-01-04

**Authors:** Ilaria Corsini, Davide Leardini, Filomena Carfagnini, Andrea Pession, Marcello Lanari

**Affiliations:** 1 Unit of Pediatrics Emergency, IRCCS Azienda Ospedaliero-Universitaria di Bologna, 40138 Bologna, Italy; ilaria.corsini@aosp.bo.it (I.C.); marcello.lanari@unibo.it (M.L.); 2 Specialty School of Pediatrics, University of Bologna, 40138 Bologna, Italy; 3 Unit of Pediatric Radiology, IRCCS Azienda Ospedaliero-Universitaria di Bologna, 40138 Bologna, Italy; filomena.carfagnini@aosp.bo.it; 4 Unit of Pediatrics, IRCCS Azienda Ospedaliero-Universitaria di Bologna, 40138 Bologna, Italy; andrea.pession@unibo.it

**Keywords:** massive pericardial effusion, pediatric emergency medicine, swinging heart sign

## Abstract

Pericardial effusion is rare in pediatric patients and is characterized by a variable clinical presentation. Mild symptoms may be present despite severe effusion. We here report the case of a patient with massive pericardial effusion with mild clinical presentation. Our case points out the need not to exclude this diagnosis in patients with mild general impairment. This clinical suspicion can be lifesaving.

## 1. Introduction

Pericardial effusion (PE) is defined as the evidence of excessive fluid within the pericardial layers and represents a rare finding in pediatric patients [1]. PE can be the symptom of the presence of several underlying pathological conditions, both inflammatory or non-inflammatory, and can include infective pericarditis, cardiac or neoplastic alterations, and anorexia nervosa [2,3,4,5]. However, a precise diagnosis often cannot be defined. PE represents a diagnostic challenge for pediatricians, considering that clinical features of children and adolescent with this condition is highly variable and can range from an incidental finding to a life-threatening emergency [6,7]. Moreover, signs and symptoms hardly can be correlated with the severity of the effusion, and thus, pediatricians of a pediatric Emergency Room (ER) may be misled by the variable clinical presentation [8]. Radiological findings can help define the exact diagnosis, but a correct clinical suspicion is essential. We here report the case of a pediatric patient presenting to the ER with a radiological confirmed massive PE but with a very mild clinical presentation.

## 2. Case Presentation

A 14-year-old girl presented at the pediatric ER with a 20-day history of mild fatigue and a two-day history of spontaneous stinging pain in the left side of the neck. The latter resembled a skeletal muscular pain, which was not modified by active or passive movements. The patient was otherwise healthy with an adequate weight, and she did not suffer from any cardiac or pulmonary diseases. One month before she had rhinitis and low-grade fever, and she was treated with azithromycin on the indication of the family pediatrician, with a limited response. For the rhinitis, a real-time polymerase chain reaction for SARS-CoV-2 on a nasopharyngeal swab was performed and resulted negative. Blood pressure was 110/70 mmHg, pulse rate was 90/min, and capillary refill time was lower than 2 s. Neither jugular venous distension nor pulsus paradoxus were present. On physical examination, the patient was asthenic with pain on palpation in the left side of the neck. Cardiac auscultation revealed slightly muffled heart sounds, but no edema or other signs of heart failure were found. Blood tests were normal except for a slight increase of c-reactive protein (CRP) (full results are reported in Table 1).

**Figure 1:**
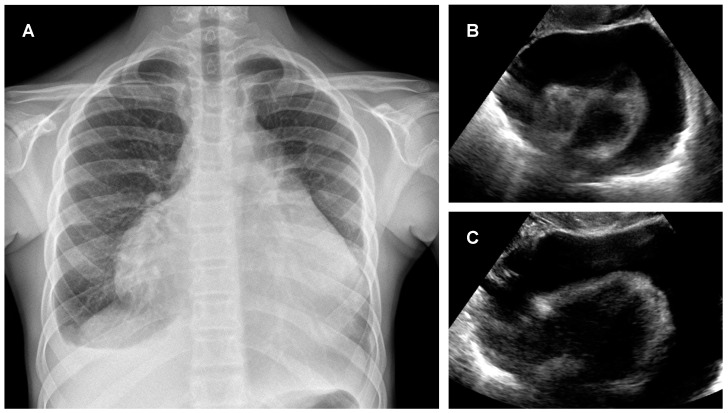
Massive pericardial effusion in a 14-year-old girl. A chest X-ray shows an abnormal cardiac silhouette with an increased cardiothoracic ratio (0.8) and bilateral pleural effusion (**A**). Echocardiography shows massive pericardial effusion with “swinging heart” signs (**B**,**C**).

Considering the astheny and the neck pain, in the absence of any reported trauma and an increase in CRP, a chest X-ray was performed. This revealed a marked enlargement of the cardiac outline with an increased cardiothoracic ratio (CTR = 0.8) and the presence of conspicuous bilateral pleural effusion resulting in pulmonary atelectasis (Figure 1, Panel A). In consideration of this, to further investigate the cardiac involvement, a point-of-care echocardiography was performed. A massive PE with the ‘swinging heart’ sign and the diastolic collapse of the free right ventricular wall was found (Figure 1, Panel B, C). The separation of the pericardial layers was 50 mm, and the diameter of the suprarenal inferior vena cava (IVC) was 26 mm. A Doppler study revealed a non-collapsible IVC during inspiration but no evidence of increased tricuspid valve inflow. Although the patient was hemodynamically stable and no signs of hypotension were found, to prevent cardiac tamponade considering the entity of the effusion, an ultrasonography-guided needle pericardiocentesis was also performed in urgency by subxiphoid puncture. From the drainage, 1500 mL of bloody fluid was removed. At the end of the procedure, no residual effusion was detectable at echocardiographic control. The culture of the pericardial fluid was negative for bacteria. Morphological and cytometric analysis on the fluid showed hematic and fibrin material, some small lymphocytes (with B CD20 subset more represented), abundant macrophages (CD68PGM1), and granulocytes. No evidence of the lymphoproliferative process was found by the fluid analysis, and malignancies were excluded considering the result of the chest X-ray and blood tests. After the intervention, pain resolved as well as fatigue in the following days. After one week the patient was discharged. To uncover the etiology of PE, IgG, and IgM for parvovirus B19, adenovirus, herpes virus 6, influenza virus, parainfluenza virus, cytomegalovirus, Epstein–Barr virus, Enterovirus, and Mycoplasma pneumonia tests were performed but resulted negative. Stool cultures resulted negative. Moreover, real-time PCR for SARS-CoV-2 on a nasopharyngeal swab performed in the ER resulted negative. The patient was also tested for autoimmunity, specifically for antinuclear antibodies, anti-mitochondrial antibodies, anti-double-stranded DNA antibodies, anti-Jo-1 antibodies, anti-Ro (SS-A) and anti-La (SS-B) antibodies, and rheumatoid factor. Thus, in the absence of any other suggestive symptoms, systemic lupus erythematosus, scleroderma, Sjogren’s syndrome, and rheumatoid arthritis were excluded. The patient was also tested for tuberculosis, using tuberculin skin test and interferon-gamma release assays, with negative result. Thus, a final diagnosis of an idiopathic polyserositis was made. In the following 16 months, the patient did not experience any recurrence of the disease and is now performing regular periodic clinical and radiological assessments.

## 3. Discussion

We report here on the case of a pediatric patient presenting to our ER with a massive PE despite few accompanying symptoms. An evacuative pericardiocentesis was performed, as is mandatory to prevent cardiac tamponade. PE represents, in fact, a diagnostic challenge for pediatricians because, often, a non-concordance between clinical and radiological signs can be found [7,9]. Our patient presented a huge amount of fluid with a mild clinical presentation. The separation of the patient’s pericardial layers was 50 mm, and current guidelines consider a “large” PE when this separation exceeds 20 mm [10]. Moreover, echocardiography showed the well-known “swinging heart” sign. In fact, while radiological findings were highly suggestive of massive PE, clinical signs of presentation were mild, according to standard classifications [11]. For instance, symptoms in adolescents usually include dyspnea, chest discomfort, peripheral edema, severe fatigue, and impaired general conditions [12].

Other authors have reported similar cases in which dramatic PE presented with mild symptoms [9]. These reports also underline that it is important to suspect cardiac tamponade when patients have hemodynamic compromise regardless of the amount of PE. Conversely, other authors reported the opposite association, namely, cardiac tamponade with small radiologically confirmed PE [7]. Interestingly, these authors reported the case of a patient with small PE causing cardiac tamponade because of a rapid accumulation of fluid within the pericardial layers. Time of fluid accumulation may represent a critical factor in determining the symptoms and thus should always be considered. In our case, the likely slow onset of PE, considering the 20-day history of fatigue, led to mild presentation despite the dramatic amount of PE. Surprisingly, the patient later revealed that she had gone on vacation the week before presentation to the ER, and she had been hiking without any impairments.

In our case, the diagnosis was confirmed by point-of-care echocardiography, which represents an evolving field across all disciplines of pediatric practice, and particularly in the context of pediatric emergency care [13]. The larger availability and portability of ultrasound makes a diffusion of this diagnostic support now possible. On the other side, non-radiologist pediatricians should deal with a new and evolving field. This should prompt specific studies to define the precise role of point-of-care in pediatric emergency care.

As previously mentioned, PE can be caused by several underlying diseases, both inflammatory and non-inflammatory, even though a definite cause may be difficult to establish in many patients. Most cases of PE in pediatric and adolescent patients were reported in the context of eating disorders [14]. Our patient did not present such disorders, and all the performed examinations resulted negative. We were, thus, unable to define an etiological diagnosis; however, the finding of rhinitis and low-grade fever some weeks before the presentation may suggest a viral infection without signs of pericarditis or a reactive polyserositis [15].

## 4. Conclusions

In conclusion, we presented the case of a patient with massive PE but with mild clinical presentation. Pediatricians should consider the non-concordance of clinical and radiological signs in evaluating patients with suspected PE; thus, in the case of mild general impairment, PE should not be excluded. Moreover, the estimated timing of symptom onset should be considered in evaluating the clinical impact of PE. Clinical suspicion can be confirmed by point-of-care echocardiography even by non-radiologist pediatricians [16]. This clinical suspicion, in our case, has been lifesaving.

## Figures and Tables

**Table 1:** Laboratory findings. WBC: white blood cells; RBC: red blood cells; Hb: hemoglobin; MCV: mean corpuscular volume; PLT: platelets; Na: sodium; K: potassium; AST: aspartateaminotransferase; ALT: alanine aminotransferase; LDH: lactate dehydrogenase; PT: prothrombin time; PTT: partial thromboplastin time; TSH: thyroid-stimulating hormone; CRP: c-reactive protein.

Exam	Result at Presentation	Normal Range
WBC (cells/mL)	11,780	4500–11,400
Neutrophils (%)	76	37–77
Lymphocytes (%)	16	20–47
Monocytes (%)	7	1–8
Eosinophils (%)	1	0–5
Basophils (%)	0	0–2
RBC (cells/mL)	4,700,000	3,900,000–5,150,000
Hb (g/dL)	12.4	12.0–15.4
MCV (fL)	79	78–93
PLT (cells/mL)	400,000	150,000–450,000
Na (mmol/L)	138	136–145
K (mmol/L)	4.7	3.5–5.3
Glucose (mg/dL)	128	60–110
AST (U/L)	21	<60
ALT (U/L)	34	<45
LDH (U/L)	191	<248
Albumin (g/L)	35.1	35.0–50.0
Total proteins (g/dL)	6.1	5.7–8.0
Amylase (U/L)	33	28–100
Lipase (U/L)	14	7–39
PT	1.2	<1.2
PTT	0.95	0.82–1.23
TSH (microU/mL)	3.43	0.25–4.50
Fibrinogen (mg/dL)	313	150–400
CRP (mg/dL)	1.1	<0.5

## Data Availability

Not applicable.
